# A CBCT Evaluation of Midpalatal Bone Density in Various Skeletal Patterns

**DOI:** 10.3390/s21237812

**Published:** 2021-11-24

**Authors:** Jong-Moon Chae, Leah Rogowski, Suchita Mandair, R. Curtis Bay, Jae Hyun Park

**Affiliations:** 1 Department of Orthodontics, School of Dentistry, University of Wonkwang, Wonkwang Dental Research Institute, Iksan 54538, Korea; jongmoon@wku.ac.kr; 2 Postgraduate Orthodontic Program, Arizona School of Dentistry & Oral Health, A.T. Still University, Mesa, AZ 85206, USA; sa202251@atsu.edu (L.R.); smandair55@gmail.com (S.M.); 3 Department of Interdisciplinary Health Sciences, A.T. Still University, Mesa, AZ 85206, USA; cbay@atsu.edu; 4 Graduate School of Dentistry, Kyung Hee University, Seoul 130-701, Korea

**Keywords:** bone density, midpalatal suture, skeletal pattern, maxillary expansion

## Abstract

The purpose of this study was to evaluate midpalatal bone density (BD) by using cone-beam computed tomography (CBCT) according to gender, age, and vertical and horizontal skeletal patterns. CBCT images from 126 subjects (64 females and 62 males) were reoriented and analyzed in order to attain BD values at the midpalatal suture. Four age groups were used for classification (adolescence, 10 ≤ early < 14 years, 14 ≤ middle ≤ 17 years, and 17 < late ≤ 21 years; adult > 21 years). Vertical skeletal pattern categories were differentiated by the Frankfort horizontal line to mandibular plane angle (hypodivergent < 22°, 22° ≤ normovergent ≤ 28°, and 28° < hyperdivergent). Horizontal skeletal pattern differentiation was defined by ANB angle (Class III < 0°, 0° ≤ Class I ≤ 4°, and 4° < Class II). Females showed significantly higher BD than males (*p* < 0.001). As age increased, BD increased significantly (*p* < 0.001). There were no significant differences between vertical skeletal patterns. Class II showed significantly less BD than Class III (*p* < 0.05). With this information, clinicians can better understand BD trends of the midpalatal suture and, thus, better understand our patient’s anatomy and potential hurdles in successful treatment.

## 1. Introduction

In order to better understand our patients’ potential treatment limitations, a thorough understanding of the anatomical variations in the midpalatal suture is critical. Studies have been conducted to investigate bone density before and after palatal expansion. For clinicians to deliver these benefits, understanding the midpalatal suture’s maturation and anatomical variations between patients is vital. A better understanding of the bone density (BD) trends of the midpalatal suture could potentially predict which patients may be more resistant to suture opening.

A proper understanding of variations in sutural bone density can help clinicians predict candidates at risk of experiencing undesirable effects of treatment affecting the midpalatal suture, including dentoalveolar expansion. Some of these undesirable side effects include loss of periodontal attachment level [1,2], buccal fenestrations [3], and root resorption [4]. Research has shown that effective opening of the midpalatal suture with rapid maxillary expansion (RME) in prepubertal subjects is associated with a significant decrease in sutural BD [5]. Identifying increased suture BD can assist in determining which expander type or expansion protocol should be used. Successful skeletal expansion may be achieved with a conventional expander in certain age groups, negating the need for a mini-implant assisted RME (MARME) or surgery.

The introduction of cone-beam computed tomography (CBCT) in orthodontics has provided diagnostic value in helping to determine expansion protocols for patients. The use of CBCT has allowed better visualization and quantitative assessment of the palate [6], particularly in relation to horizontal and vertical growth patterns, which helps in the decision of whether to use conventional or surgically assisted maxillary expansion. The purpose of this study is to measure mean BD values of the midpalatal suture in order to provide a more accurate estimation of the midpalatal response to expansion therapy as related to gender, age categories, and vertical and horizontal skeletal patterns.

## 2. Materials and Methods

### 2.1. Subjects

A sample size of 126 achieves 85% power to detect an r-squared of 0.10 (a medium effect size) attributed to four independent variable(s) using an F-Test with a significance level (alpha) of 0.05. Existing routine diagnostic CBCT images of a total of 126 selected patients (62 males and 64 females) were utilized for midpalatal BD analysis after institutional review board (IRB) approval. The CBCT (PSR 9000N; Asahi Alphard Vega, Kyoto, Japan) images were taken from the archives (from May 2007 to May 2016) of the orthodontic department at the School of Dentistry, Wonkwang University, Daejeon Dental Hospital in Daejeon, South Korea. Participant exclusion criteria included a history of comprehensive orthodontic treatment, impacted teeth, dentofacial abnormalities, pathologies, skeletal asymmetry, history of periodontal disease, and missing dentition.

**Figure 1:**
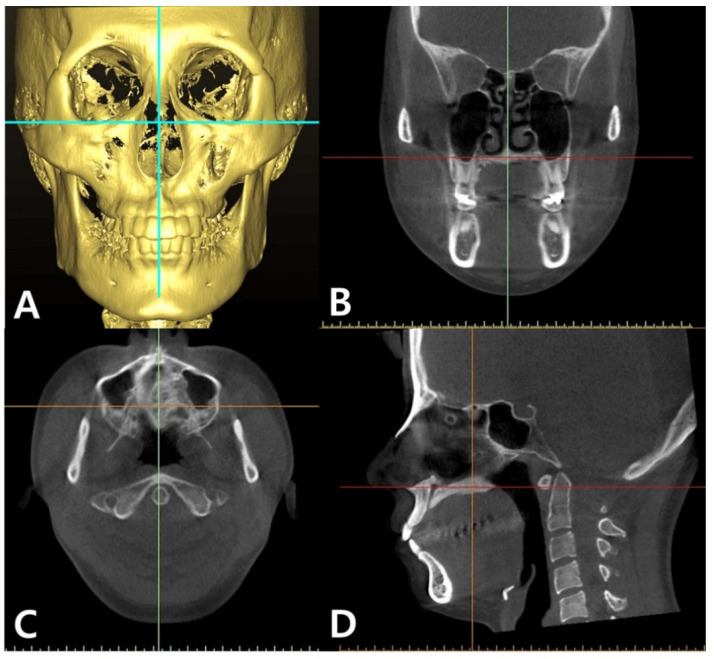
Process of cone-beam computed tomography (CBCT) image reorientation in the multiplanar orientation screen: (**A**) CBCT image was reorientated along the inferior border of the orbital rims in the frontal reconstructed view. (**B**,**C**) In the coronal and axial views, CBCT images were reoriented to allow for analysis at the midpalatal suture so that the green vertical line was positioned through the anterior nasal spine (ANS) to posterior nasal spine (PNS). (**D**) In the sagittal view, the CBCT image was rotated to confirm that the midpalatal line ran through ANS to PNS.

**Figure 2:**
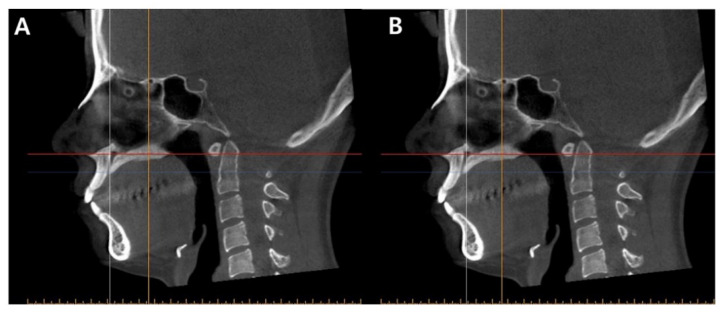
Sagittal view for bone density measurements: (**A**) Cone-beam computed tomography (CBCT) image was oriented so that the white vertical line was placed along the posterior border of the incisive foramen at an exit of the inferior canal. (**B**) The white vertical orientation line was then moved posteriorly by 1.56 mm to create the anterior limit for data collection.

**Figure 3:**
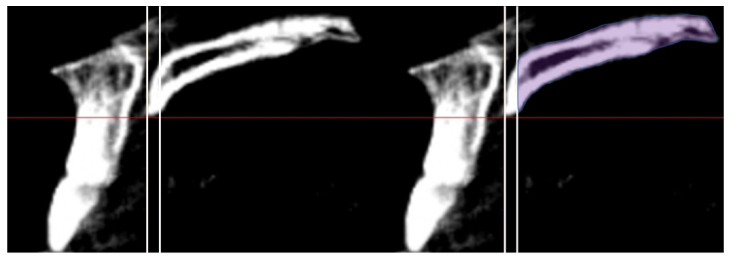
Using the “create graft volume” function, bone density was measured. The area was selected manually along the bone border.

BD dimensions were measured by using Simplant software (Dentsply Sirona, York, PA, USA). The CBCT images were first reoriented along the inferior border of the orbital rims in the reconstructed frontal view (Figure 1A). In the coronal and axial views, CBCT images were reoriented to allow for analysis at the midpalatal suture. The CBCT images were then orientated so that a green vertical line could be positioned through the anterior nasal spine (ANS) relative to the posterior nasal spine (PNS) in order to mark the midpalatal suture (Figure 1B,C). The images were rotated in the sagittal view to confirm that the midpalatal line ran through ANS to PNS (Figure 1D). Once this orientation was complete, in the sagittal view, a vertical line was placed along the posterior border of the incisive foramen at an exit of the inferior canal. (Figure 2A). The vertical orientation line was moved posteriorly by 1.56 mm to create the anterior limit for data collection (Figure 2B). This ensured that the anterior limit was completely out of the incisive canal and into the palatal bone. BD was measured by using the “create graft volume” function; an icon was pushed on the software to calculate the predetermined bone volume and density. The area was selected manually along the bone border. The software provided a mean BD value in Hounsfield units (HU) for each area of the midpalatal suture selected. BD and standard deviation (SD) measurements were then gathered from the sagittal slices (Figure 3).

Gender, age, and vertical and horizontal skeletal categories were used to predict BD. Four age groups were used (adolescence, 10 ≤ early < 14 years, 14 ≤ middle ≤ 17 years, and 17 < late ≤ 21 years; adult > 21 years). For vertical skeletal pattern analysis, the subjects were differentiated by the Frankfort horizontal line to the mandibular plane angle (hypodivergent < 22°, 22° ≤ normovergent ≤ 28°, and 28° < hyperdivergent). For horizontal growth pattern differentiation, the subjects were divided by the ANB angle (Class III < 0°, 0° ≤ Class I ≤ 4°, and 4° < Class II).

### 2.2. Statistical Analysis

Two researchers (C.S. and J.S.) measured BD and completed data collection independently for 10 randomly chosen participants in order to assess reliability. Both the intra-rater and inter-rater intraclass correlation coefficients (ICC) showed excellent reliability. Using a random, two-way, single measurement model for consistency, the median ICCs were calculated across 65 slices. The median intra-rater ICC for Rater 1 was 0.90, and for Rater 2, it was 0.96. The median inter-rater ICC was 0.93.

Descriptive statistics are provided as means and standard deviations. For inferential tests, marginal means, mean differences, and 95% confidence intervals are provided. A generalized linear model approach was used to evaluate the differences in mean BD across age categories, gender, and vertical and horizontal skeletal patterns. All predictor variables were entered simultaneously so that the unique contribution of each to BD could be estimated. Bonferroni corrections were applied to follow-up pairwise tests, as appropriate. A trend analysis was also used to examine BD by age group. Statistical significance for all tests was set at alpha = 0.05, two-tailed. Analysis was conducted using IBM SPSS software ver. 24.0 (IBM Corp., Armonk, NY, USA).

## 3. Results

Individuals numbering 126 were included in the final analysis (Table 1). The preliminary tests of normality (Kolmogorov–Smirnov) of BD for the various categories (gender, age categories, and vertical and horizontal skeletal patterns) identified no assumption violations. Means and standard deviations for midpalatal BD by predictor categories are provided in Table 2 and Table 3. Pairwise comparisons of BD for each of the predictor categories, along with Bonferroni-corrected *p*-values for pairwise comparisons, are provided in Table 4. In the first column, “Variables,” the direction of the subtraction is indicated by the order of the variables listed in order to derive the difference score. For example, “Male vs. Female” means that the female score was subtracted from the male score.

Females demonstrated a significantly higher mean BD at the midpalatal suture than males. The trend analysis for BD by age group was significant (*p* < 0.001), showing increasing BD with increasing age. As observed, BD differed significantly between the age categories, while BD did not differ significantly across vertical growth patterns. For vertical growth patterns, Class III demonstrated significantly higher BD than Class II (Table 4).

## 4. Discussion

Anatomical variations in patients have the potential to cause the failure of rapid maxillary expansion. Although this study does not evaluate sutural failure, it is important to evaluate and understand the anatomical variations in a patient’s midpalatal bone density because sutural expansion failure is not an unusual occurrence in adolescent and young adult patients [7]. Studies have shown significant variability in the fusion of the midpalatal suture. Persson and Thilander reported that midpalatal fusion occurs from ages 15 to 19 [8], but other studies have shown patients with no signs of sutural fusion at the ages of 27, 32 [8], 54 [9], and 71 [10,11]. It is essential that clinicians understand sutural maturation and sutural opening in order to mitigate negative effects, such as periodontal defects [1,2,3] created by dentoalveolar tipping. The unpredictability of true suture opening has resulted in many modalities for accurately assessing midpalate suture’s maturation. Some methods used to evaluate palatal maturation include hand-wrist radiographs, cervical vertebral maturation stages (CVMS), occlusal radiographs, BD ratios at the palate, and a five-stage maturational analysis of the midpalatal suture [12,13,14,15,16,17]. The five-stage palatal maturational classification by Angelieri et al. [17] was one of the first techniques to directly examine the palate to understand its maturation. The study evaluated the morphology of the midpalatal suture [11]. A review of previous palatal maturation literature suggests that accurate analysis of palatal suture maturation should be considered before treatment planning [12].

One CT study concluded that effective opening of the suture with RME in prepubertal subjects is associated with a significant decrease in sutural BD [5]. Therefore, an increased fusion of the suture results in increases in palatal BD and, thus, an increase in resistance to expansion. Additionally, many studies have suggested that midpalatal suture BD is one of the most important factors in determining the resistance of the midpalatal suture expansion forces [10,12,18,19]. The present study consequently aimed to evaluate the palate itself in order to best understand BD changes across several variables. Evaluation of the midpalatal BD, rather than a single rectangular slice or area in the palate as was performed in previous studies [12,20], further helps to account for the variation that exists in the manner the suture closes [13].

Since CBCT is widely used and easily accessible to orthodontists due to its routine use in orthodontic diagnosis and treatment, it was thought to be the most beneficial medium for analysis in this study. However, minimal radiation exposure should be considered. Although CTs are the gold standard of BD analysis, the relationship between CT and CBCT values has been very consistent when evaluating BD values, allowing CBCT to be considered an alternative diagnostic tool [6]. CBCT images are more affordable, emit lower radiation dosages, and allow visualization of the midpalate suture without overlapping anatomical structures [6,19]. Despite the benefits of CBCT for BD analysis, there have been concerns about its reproducibility. CBCT gray BD values vary from scanner to scanner [21]. In this study, however, all radiographic images were taken from the same CBCT machine with the same settings and exposure protocol, reducing variation in gray BD values. Using images from a single CBCT allows for the analysis of relative BD values that can be used to identify specific trends [21]. Therefore, future studies are essential in order to derive conversion factors between CBCT and CT to obtain absolute values.

This study’s results demonstrated that females have a significantly higher midpalatal suture BD than males. These results coincide with those by Han et al. [22] and Moon et al. [23], who reported that females have higher palatal cortical bone densities than males. Additionally, in this study, as age increased, palatal BD also increased at the midpalatal suture. The increase in BD with age was in line with the theory that a proportional relationship exists between BD and its resistance to fracture. The midpalatal suture BD may be the most reliable explanation for maxillary resistance to expansion with age [20]. The statistically significant difference in BD values between early and late adolescence signifies that there is a substantial increase in interdigitation and, therefore, maturation at the suture during middle adolescence. In a systematic review conducted by Liu et al. [19], significant differences were found between age groups, with the middle-aged group exhibiting the highest BD. This study also revealed that the mean BD value was the greatest for Class III individuals. This can be a notable finding for patients in middle adolescence verging on late adolescence. For Class III late adolescent patients, expansion by surgical options or skeletal anchorage supported devices may be more beneficial in creating true splitting of the palatal suture for expansion at the skeletal level.

According to Franchi et al.’s study [5], it was possible to open sutures with conventional expansion therapy when midpalate BDs ranged from 563.3 to 741.7 HU (as measured by CT). Although expansion is viable over a range of densities, greater resistance midpalatally creates a greater probability of dentoalveolar effects. This study further clarifies the necessity to understand when significant variance in midpalatal BD can potentially result in the detrimental impacts of expansion. BD was 21% less dense in early adolescence than in late adolescence. BD was 17% less dense in males than in females, and Class II individuals had approximately 13% less dense bone at the midpalatal suture. These variations demonstrate that adverse side effects are less likely in younger Class II and male patients with expansion therapy. When providing expansion therapy, factors such as posterior alveolar bone housing width and gingival biotype should also be evaluated along with gender, vertical and horizontal growth patterns and age during treatment planning in order to provide the best possible patient outcomes with minimal adverse effects [24,25].

Although this study shows general trends relative to palatal BD, for critical cases between adolescents and young adults, classifications reported by Angelieri et al. [17] might offer further insight in determining expansion treatment modalities. Future directions for evaluation of BD using this method include evaluating subjects with Angelieri et al.’s [17] five-stage classification of midpalatal suture morphology. Evaluating BD in different antero-posterior regions of the palate could also provide insight into trends across genders, age groups, and different skeletal patterns, which could influence orthodontic treatment decisions. Finally, increasing the number of subjects evaluated could help determine whether a specific age demonstrates a significant increase in palatal BD, suggesting a chronological age limit to conventional rapid palatal expansion techniques.

## 5. Conclusions

1. Females showed significantly higher midpalatal BD than their male counterparts.
2. Late adolescence individuals showed significantly less BD at the midpalatal suture than did individuals in early adolescence.
3. Class II skeletal individuals showed significantly less midpalatal BD than Class III individuals.

## Figures and Tables

**Table 1:** Sample distribution (number).

Variables			Gender		Age (y)				Mandibular Plane Angle (MPA)			ANB Angle		
			M	F	Adolescence			Adult	Hypodivergent	Normover-gent	Hyperdivergent	Class III	Class I	Class II
					Early	Middle	Late							
					10 ≤ y < 14	14 ≤ y ≤ 17	17 < y ≤ 21	y > 21	MPA < 22°	22° ≤ MPA ≤ 28°	MPA > 28°	ANB < 0°	0° ≤ ANB ≤ 4°	ANB > 4°
Gender	Male		62		13	21	13	15	19	23	20	23	24	15
	Female			64	18	13	19	14	22	20	22	15	32	17
Age		Early	13	18					9	11	11	4	15	12
	Adolescence	Middle	21	13					11	12	11	7	16	11
		Late	13	19					10	11	11	16	12	4
	Adult		15	14					11	9	9	11	13	5
MPA	Hypodivergent		19	22	9	11	10	11				14	19	8
	Normovergent		23	20	11	12	11	9				15	18	10
	Hyperdivergent		20	22	11	11	11	9				9	19	14
ANB	Class III		23	15	4	7	16	11	14	15	9			
	Class I		24	32	15	16	12	13	19	18	19			
	Class II		15	17	12	11	4	5	8	10	14			
Total (each)			126		31	34	32	29	41	43	42	38	56	32

**Table 2:** Midpalatal bone density (BD) according to gender and age (HU).

	Means (Standard Deviations)					
Variables	Gender		Age (y)			
	Male (n = 62)	Female (n = 64)	Early: 10 ≤ y < 14 (n = 31)	Middle: 14 ≤ y ≤ 17 (n = 34)	Late: 17 < y ≤ 21 (n = 32)	Adult: y > 21 (n = 29)
BD	549.33 (113.06)	657.25 (110.93)	529.05 (96.45)	561.13 (124.57)	662.09 (112.58)	679.90 (96.99)

HU; Hounsfield units.

**Table 3:** Midpalatal bone density (BD) according to skeletal patterns (HU).

	Means (Standard Deviations)					
Variables	Mandibular Plane Angle (MPA)			ANB Angle		
	Hypodivergent MPA < 22° (n = 41)	Normovergent 22° ≤ MPA ≤ 28° (n = 43)	Hyperdivergent MPA > 28° (n = 42)	Class III ANB < 0° (n = 38)	Class I 0° ≤ ANB ≤ 4° (n = 56)	Class II ANB > 4° (n = 32)
BD	606.93 (105.57)	586.34 (136.06)	619.65 (128.25)	633.82 (117.43)	613.90 (123.99)	551.83 (119.10)

HU; Hounsfield units.

**Table 4:** A generalized linear model is derived from comparing midpalatal bone densities (BDs) according to gender, age, and skeletal patterns.

Variables	Mean BD Difference (HU)	95% Wald Confidence Interval		*p*-Value
		Lower	Upper	
Gender				
Male vs. Female	−108.75	−140.25	−77.25	<0.001 ***
Age				
Early vs. Middle	−47.35	−96.48	1.80	0.062
Early vs. Late	−104.04	−163.76	−44.32	<0.001 ***
Early vs. adult	−121.22	−182.35	−60.09	<0.001 ***
Middle vs. Late	−56.69	−110.93	−2.46	0.037 *
Middle vs. Adult	−73.87	−129.97	−17.78	0.004 **
Late vs. Adult	−17.18	−61.50	27.14	0.447
Vertical skeletal pattern				
Hypo vs. Normo	15.74	−21.74	53.22	0.410
Hypo vs. Hyper	−28.14	−71.63	15.35	0.294
Normo vs. Hyper	−43.88	−89.67	1.90	0.065
Horizontal skeletal pattern				
Class I vs. Class II	42.92	−1.07	86.90	0.057
Class I vs. Class III	−17.39	−55.28	20.50	0.368
Class II vs. Class III	−60.31	−114.70	−5.92	0.024 *

HU; Hounsfield units. A vs. B means that the B score is subtracted from the A score. * *p* < 0.05, ** *p* < 0.01, *** *p* < 0.001.
